# Endodontic Microsurgery as a Primary Treatment for Pulp Canal Obliteration With Apical Periodontitis: Case Reports

**DOI:** 10.1155/crid/7497117

**Published:** 2026-09-01

**Authors:** Beichen Mou, Heng Tu, Jing Yang

**Affiliations:** ^1^ State Key Laboratory of Oral Diseases and National Center for Stomatology and National Clinical Research Center for Oral Diseases, Department of Cariology and Endodontics, West China Hospital of Stomatology, Sichuan University, Chengdu, China, scu.edu.cn; ^2^ Key Laboratory of Bio-Resource and Eco-Environment of Ministry of Education, College of Life Sciences, Sichuan University, Chengdu, China, scu.edu.cn

**Keywords:** apical periodontitis, bioceramic material, endodontic microsurgery, pulp canal obliteration

## Abstract

Pulp canal obliteration (PCO), commonly following dental trauma, is characterized by progressive canal calcification and may be associated with pulp necrosis and apical periodontitis (AP). Conventional root canal treatment (RCT) may be technically unfeasible or associated with a high risk of iatrogenic complications. In the case reports, we explored endodontic microsurgery (EMS) as a primary treatment strategy for managing PCO with AP. Three patients (aged 30–46 years) presented with a history of dental trauma, severe canal calcification, and radiographic evidence of apical radiolucency, with or without associated bone loss. All cases were diagnosed using cone beam computed tomography (CBCT) and managed with EMS as the primary treatment approach, combined with iRoot BP Plus for apical sealing. Clinical symptoms, including gingival abscesses, resolved following treatment, and radiographic follow‐up (up to 3 years) demonstrated resolution of periapical radiolucency and evidence of bone healing in all cases. Within the limitations of these case reports, primary EMS may represent a feasible alternative for selected cases of PCO with AP, allowing effective management of periapical pathology while avoiding the risks associated with attempting RCT in severely calcified canals.

## 1. Introduction

Pulp canal obliteration (PCO), also referred to as calcific metamorphosis, is a common pulpal sequela of dental trauma [1, 2]. It is characterized by accelerated and aberrant deposition of hard tissue within the root canal space, leading to progressive narrowing or complete radiographic disappearance of the canal lumen [1]. Clinically, affected teeth are often asymptomatic; however, a gradual yellowish discoloration of the crown and a reduced or absent response to pulp sensibility testing are characteristic findings [3]. Radiographically, partial or complete obliteration of the canal space is typically observed [1, 4]. Recent studies have reported the prevalence of PCO at 27.6% in traumatized permanent teeth [5], 12.9% in teeth with lateral or extrusive luxation [6], 42.5% following alveolar process fractures [7], and up to 22% in teeth followed for 3 years after trauma [8]. And a proportion of these cases may subsequently progress to pulp necrosis and apical periodontitis (AP) [5, 9].

For symptomatic cases of PCO with established periapical pathology, conventional root canal treatment (RCT) can be technically challenging due to the difficulty of identifying and negotiating severely calcified canals [1]. Freehand instrumentation carries a substantial risk of iatrogenic complications, including canal deviation, ledge formation, perforation, and instrument separation, which may further compromise tooth structure and prognosis [10]. Although techniques such as guided endodontics have been introduced to improve access, their clinical application remains limited, particularly in complex cases where the deviation can exceed 0.5 mm at the apical terminus [11–14].

Endodontic microsurgery (EMS) has traditionally been regarded as a second‐line intervention, primarily indicated after failure of nonsurgical RCT to resolve persistent periapical disease [15–17]. However, in cases of severe canal calcification, orthograde treatment may be impractical or associated with a high risk of iatrogenic damage [1, 10]. In contrast, EMS provides direct access to the periapical lesion, enabling thorough debridement, apical resection, and retrograde sealing under magnification. This approach allows effective elimination of apical infection while avoiding the challenges of canal negotiation and preserving coronal tooth structure [18]. In addition, modern microsurgical techniques and bioceramic materials have significantly improved the precision and predictability of surgical outcomes [19, 20]. A recent study has explored chamberless endodontic access (CEA) for the surgical management of PCO with AP, demonstrating the feasibility of a retrograde approach while preserving coronal structure [21]. However, conventional materials such as gutta‐percha were used for root‐end filling, which may have limitations in sealing ability compared with contemporary bioceramics, such as iRoot BP Plus (a bioceramic calcium silicate‐based cement), for achieving improved apical sealing [20]. Therefore, EMS may represent a rational alternative in selected cases, particularly when conventional RCT is unlikely to be successful.

The present case reports describe three cases of PCO with symptomatic AP following different traumatic etiologies, in which EMS combined with iRoot BP Plus apical sealing was used as the primary treatment approach based on clinical considerations. These reports are aimed at presenting the clinical rationale and outcomes associated with this strategy and illustrating the potential role of primary EMS as an alternative treatment option in carefully selected cases of PCO with AP.

## 2. Case Presentation

These case reports follow the 2020 Preferred Reporting Items for Case reports in Endodontics (PRICE) guidelines; a flowchart containing case details and an appropriate checklist were provided [22]. All clinical examinations, radiographic evaluations, and the surgical procedures were directly performed by a single experienced endodontic microsurgeon (J.Y.), with the support of trained clinical assistants (B.M. and H.T.).

### 2.1. Case 1

A 30‐year‐old male presented to the department of endodontics at a university hospital with a chief complaint of a persistent sinus tract adjacent to Tooth #8 for over 1 month. The patient reported a history of dental trauma to the same tooth approximately 10 years earlier. The patient was systemically healthy, a nonsmoker, and taking no regular medications.

Clinical examination of Tooth #8 revealed an intact crown without caries. A sinus tract with purulent discharge was observed on the labial alveolar mucosa. Cold testing was performed using Endo‐Frost refrigerant (Coltene/Whaledent, Langenau, Germany) spray applied to a cotton pellet, which was then placed on the tooth surface. The tooth was nonresponsive to cold and negative to palpation. It showed mild tenderness to percussion and normal mobility. Periodontal probing did not reveal isolated deep periodontal pockets.

**Figure 1:**
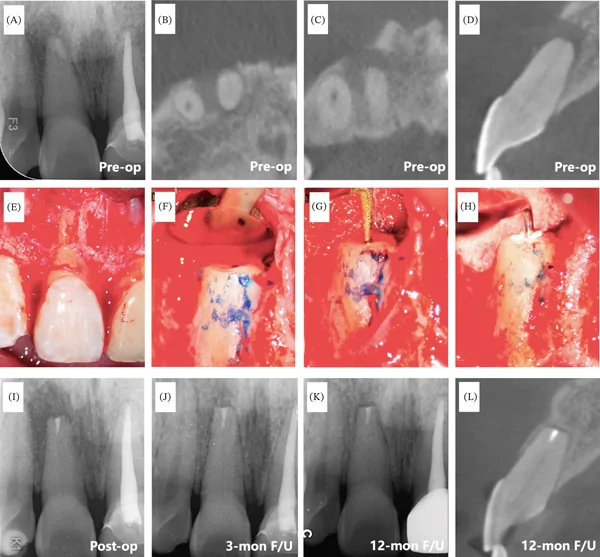
Preoperative, intraoperative, and postoperative imaging of Case 1 (Tooth #8). (A) Preoperative PA demonstrating PCO with an associated periapical radiolucency of Tooth #8. (B–D) Preoperative CBCT images revealing an indistinct root canal, a large periapical radiolucent lesion, and complete resorption of the labial cortical plate of Tooth #8. (E) Intraoperative photograph following flap reflection showing a labial bone dehiscence extending to the root apex. (F) Resection of the apical 3 mm. (G) Ultrasonic retrograde preparation to a depth of 3 mm. (H) Root‐end filling with iRoot BP Plus. (I) Immediate postoperative PA of Tooth #8. (J) Three‐month follow‐up PA showing significant resolution of the periapical lesion. (K,L) Twelve‐month follow‐up PA and CBCT image demonstrating complete periapical healing and regeneration of the labial cortical plate. CBCT, cone beam computed tomography; PA, periapical radiograph.

Periapical radiography (PA) was performed using a dental x‐ray machine (Carestream CS2100, Rochester, United States), and cone beam computed tomography (CBCT) was performed using a CBCT unit (Morita Corp., Kyoto, Japan). Radiographic examination demonstrated severe canal calcification and a large periapical radiolucency (Figure 1A–D). Notably, extensive labial bone loss was present, resulting in marked reduction of buccal cortical support around the root (Figure 1B,D). A diagnosis of PCO with AP of Tooth #8 was established.

Available treatment options, including EMS, conventional RCT, extraction, and deferral, were discussed. Given the severity of canal calcification and the extent of periapical destruction, conventional RCT was considered impractical. EMS was therefore selected as the primary treatment approach, and written informed consent for the procedure, along with permission for publication of the case and associated clinical images, was obtained from the patient.

EMS was performed under a surgical microscope (Carl Zeiss, Göttingen, Germany). Local anesthesia was achieved using 2% lidocaine with 1:100,000 epinephrine (Septodont, Brampton, Canada) and 4% articaine hydrochloride with 1:100,000 epinephrine (Acteon, Mérignac, France). A full‐thickness mucoperiosteal flap was elevated, revealing extensive labial bone dehiscence extending to the root apex (Figure 1E), consistent with Class F according to the Kim and Kratchman classification [23]. Osteotomy was performed using a high‐speed carbide bur (M15ZB, MANI, Tochigi, Japan) under copious sterile saline irrigation. Granulation tissue was curetted and submitted for histopathological examination. Following resection of 3 mm of the root apex, methylene blue (Jumpcan, Hubei, China) staining was used to inspect the resected root surface under high magnification (Figure 1F). Retrograde preparation was performed using an ultrasonic tip (B&L, Gyeonggi‐do, Korea) to a depth of 3 mm (Figure 1G). The cavity was subsequently filled with iRoot BP Plus (Innovative BioCeramix Inc., Vancouver, Canada) and finished (Figure 1H). Guided tissue regeneration was performed using a concentrated growth factor (CGF) membrane. The flap was repositioned and sutured with 5‐0 nylon (Johnson & Johnson, New Jersey, United States). PA was taken after the surgery (Figure 1I).

Histopathological analysis confirmed chronic inflammatory changes consistent with an apical granuloma. At the 3‐ and 12‐month follow‐ups, radiographic examination demonstrated substantial bone regeneration and resolution of the periapical lesion (Figure 1J–L). The patient remained asymptomatic, with no evidence of postoperative complications.

### 2.2. Case 2

A 30‐year‐old female presented to the department of endodontics at a university hospital with a recent episode of spontaneous pain in Tooth #8, which had resolved spontaneously within 3 days without medication. The patient had no systemic diseases, did not smoke, and was on no regular medications.

**Figure 2:**
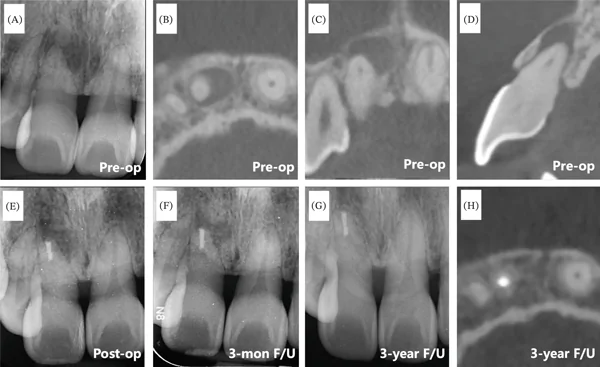
Preoperative and postoperative imaging of Case 2 (Tooth #8). (A) Preoperative PA demonstrating PCO of Tooth #8 with an associated periapical radiolucency. (B–D) Preoperative CBCT images revealing a completely obliterated mid‐root canal, an apical radiolucent lesion, and a reduced intraosseous root length of Tooth #8. (E) Immediate postoperative PA of Tooth #8. (F) Three‐month follow‐up radiograph depicting substantial resolution of the periapical radiolucency. (G,H) Three‐year follow‐up PA and CBCT image confirming complete periapical healing. CBCT, cone beam computed tomography; PA, periapical radiograph.

Clinical examination revealed crown discoloration, negative responses to cold and palpation, positive percussion sensitivity, and pain on biting. No sinus tract formation or periodontal probing abnormalities were observed. Radiographic and CBCT examinations demonstrated a periapical radiolucent lesion, pulpal canal calcification, and reduced intraosseous root length (Figure 2A–D). Based on clinical and radiographic findings, a diagnosis of PCO associated with AP was established for Tooth #8.

Treatment alternatives such as EMS, conventional RCT, extraction, and observation were fully explained. Given severe canal calcification with non‐negotiable canal anatomy and limited intraosseous root length, conventional RCT was considered to have limited predictability in achieving adequate canal disinfection. After comprehensive evaluation, EMS was selected as the primary treatment approach to directly address the periapical pathology and ensure controlled management under constrained anatomical conditions. Written informed consent was obtained from the patient for treatment and publication of this case and accompanying clinical images.

EMS was performed on Tooth #8 following established microsurgical protocols as previously described. Intraoperatively, no evident buccal bone fenestration was observed, corresponding to the Kim and Kratchman EMS Class C classification [23]. PA was obtained immediately after surgery to confirm procedural quality (Figure 2E).

At the 3‐month and 3‐year follow‐ups, radiographic evaluation demonstrated significant reduction of the periapical radiolucency and progressive bone regeneration with increased trabecular density (Figure 2F–H). The patient remained asymptomatic throughout the follow‐up period, with no postoperative complications or evidence of disease recurrence.

### 2.3. Case 3

A 46‐year‐old female was referred to the department of endodontics at a university hospital, presenting with persistent discomfort and occlusal dysfunction in the mandibular left posterior region for 5 months. The dental history of this region was complex, including a cantilever fixed partial denture placed more than 10 years earlier, extraction of Tooth #21 due to a gingival abscess 9 months previously, and subsequent splinted crown restoration 5 months prior. None of the involved teeth had received prior endodontic treatment. The patient was systemically healthy, a nonsmoker, and not taking any medications.

**Figure 3:**
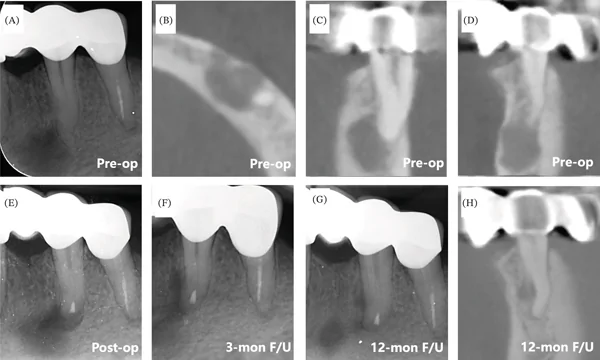
Preoperative and postoperative imaging of Case 3 (Tooth #21). (A) Preoperative PA demonstrating root curvature and a mesial radiolucency associated with Tooth #21. (B–D) Preoperative CBCT images revealing complete radiographic obliteration of the middle and apical root canal, an extensive mesial radiolucent lesion, and severe resorption of the buccal cortical plate of Tooth #21. (E) Immediate postoperative PA of Tooth #21. (F) Three‐month follow‐up radiograph depicting significant resolution of the periapical radiolucency. (G,H) Twelve‐month follow‐up PA and CBCT image confirming complete healing of the periapical lesion, with only a small residual radiolucency remaining on the mesial aspect. CBCT, cone beam computed tomography; PA, periapical radiograph.

Clinical examination revealed tenderness to percussion and palpation in Tooth #21, a negative response to cold testing, with no detectable sinus tract and physiological tooth mobility. Radiographic and CBCT evaluation demonstrated that the middle and apical portions of the canal were indistinct or absent and exhibited pronounced curvature. A nearly complete loss of the buccal cortical plate was observed, accompanied by a large radiolucent area involving the periapical and mesial regions (Figure 3A–D).

A diagnosis of PCO associated with AP in Tooth #21 was established. Options including EMS, conventional RCT, extraction, and watchful waiting were discussed. The patient expressed a strong preference to retain the existing restoration, and the marked curvature of the apical canal further reduced the likelihood of successful orthograde negotiation. EMS was therefore chosen as the primary intervention, with written informed consent obtained from the patient for both the treatment and the publication of the case and accompanying clinical images. Intraoperatively, complete buccal bone dehiscence was identified, corresponding to Class F according to the Kim and Kratchman EMS classification [23].

Postoperative PA confirmed adequate root‐end management (Figure 3E). At the 3‐ and 12‐month follow‐ups, radiographic evaluation demonstrated substantial bone regeneration with marked reduction of radiolucency, and the patient reported complete resolution of symptoms and functional restoration (Figure 3F–H).

## 3. Discussion

These case reports describe three patients with PCO and advanced AP involving different age groups and tooth types, all with a history suggestive of dental trauma. The etiological backgrounds varied, including a clearly documented traumatic event (Case 1), crown discoloration indicative of previous injury (Case 2), and long‐term occlusal stress potentially contributing to pulpal changes (Case 3). CBCT imaging showed a diffusely blurred canal with faint residual patency in Case 1, complete disappearance of the mid‐root canal in Case 2, and total obliteration of the middle and apical thirds in Case 3. Additional findings included labial or buccal bone loss (Cases 1 and 3) and external root resorption (Case 2). All three cases achieved favorable healing outcomes following a standardized microsurgical protocol involving 3‐mm root‐end resection and retrograde sealing with iRoot BP Plus. Clinical symptoms resolved, and radiographic evidence of progressive bone healing was observed within 12 months in all cases. Stable outcomes were maintained at the 3‐year follow‐up in the one case with available data.

The pathogenesis of PCO in the reports aligns with its association with dental trauma, though the triggers varied. While Case 1 follows the typical slow progression postluxation, Case 3 suggests that chronic mechanical stress from occlusal overload may also induce similar calcific changes, a factor less frequently highlighted than acute trauma. Although pulp necrosis is considered uncommon in teeth with trauma‐related PCO, it may develop during long‐term follow‐up, particularly when periapical disease becomes evident. Therefore, endodontic intervention should be considered based on clinical and radiographic findings rather than PCO alone [5, 9].

The clinical decision to bypass conventional RCT in these cases was driven by the high risk‐to‐benefit ratio of orthograde negotiation [1, 10, 18]. Unlike the generalized challenges mentioned in the literature, specific anatomical hurdles here—such as the complete mid‐root obliteration in Case 2 and the pronounced apical curvature in Case 3—rendered nonsurgical access likely to result in iatrogenic damage. Although guided endodontics has significantly improved the management of calcified canals, it remains technique‐sensitive and may be limited by restricted mouth opening, inadequate interocclusal space, extensive apical curvature, severe cortical bone destruction, or economic considerations [13, 14, 24]. In such situations, primary EMS may represent a predictable alternative that avoids extensive coronal dentin removal while providing direct access to the apical pathology.

Our cases confirmed this advantage. We observed several distinct benefits. In Case 1, the existing labial bone defect provided a natural surgical window; in Case 2, it preserved the integrity of a short intraosseous root; and in Case 3, it avoided the removal of a functional prosthetic restoration. In all three cases, this approach avoided intraradicular complications and resulted in favorable healing outcomes. These findings support the consideration of EMS as a primary treatment option in carefully selected cases where conventional RCT is unlikely to succeed [15].

Nevertheless, careful case selection remains essential. Primary EMS should not be considered a routine substitute for conventional RCT but may be appropriate when orthograde canal negotiation is unlikely to succeed or carries a substantial risk of iatrogenic complications.

With these selection criteria in mind, we propose the following specific scenarios in which primary EMS may be considered a rational first‐line strategy for PCO with AP: when the canal is severely calcified and radiographically untraceable (as in Case 2, where the middle third was completely obliterated), when pronounced root curvature further complicates orthograde negotiation (as in Case 3), when the intraosseous root length is reduced and preservation of remaining tooth structure is paramount (Case 2), when the patient wishes to retain an existing prosthetic restoration (Case 3), and when extensive resorption of the buccal or labial cortical plate provides a favorable surgical window (Case 1). In all such situations, preoperative CBCT evaluation is indispensable for identifying anatomical characteristics favorable for this treatment approach. These characteristics include the extent of calcification, the presence of curvature, and the status of the cortical bone, thereby guiding appropriate case selection for this minimally invasive, outcome‐predictable approach [15, 25].

According to the Kim and Kratchman classification, Case 2 was categorized as Class C, whereas Cases 1 and 3 were classified as Class F, indicating extensive buccal bone loss [23]. Such defects are considered challenging and may benefit from adjunctive regenerative approaches. In the reports, CGF membranes were applied to support guided tissue regeneration. The observed regeneration of the buccal cortical plate in Cases 1 and 3 may be partly attributable to the adjunctive use of CGF, which provides a bioactive scaffold for guided tissue regeneration [26, 27]; however, its precise contribution remains speculative in the absence of a control group.

The presence of a sinus tract, apical root resorption, and large periapical lesions indicated advanced periapical disease in the three cases. Given the severe canal obliteration, effective canal shaping and chemical disinfection were challenging [28–30]. The favorable outcomes observed in the reports may be related to the standardized surgical protocol involving 3‐mm root‐end resection and 3‐mm retrograde preparation. Previous studies have demonstrated that resection of the apical 3 mm removes the majority of apical ramifications and lateral canals, thereby reducing bacterial persistence [15, 23]. Retrograde preparation to a similar depth, followed by sealing with iRoot BP Plus, may provide an effective apical barrier and reduce microleakage. iRoot BP Plus is a bioceramic material that undergoes a hydration reaction with slight expansion upon contact with dentin, facilitating tight interfacial bonding [31]. The setting reaction generates hydroxyapatite, contributing to long‐term seal stability. Compared with MTA, iRoot BP Plus offers shorter setting time and reduced discoloration potential, making it particularly suitable for esthetically sensitive regions [20, 32].

This study has several limitations. First, the small sample size and case‐report design limit the generalizability of the findings. In addition, because these cases were treated by an experienced endodontic microsurgeon at a tertiary referral center, the findings may not be directly generalizable to all clinical settings. The number of postoperative visits was also limited because of various personal and geographic factors. Moreover, long‐term follow‐up beyond 12 months was available for only one of the three cases. Although the 3‐year outcome in that case remained stable, the limited long‐term data warrant cautious interpretation. Longer follow‐up periods are therefore needed to evaluate the long‐term stability and predictability of the treatment outcomes. Larger prospective studies with control groups are required to validate these findings. Comparative studies evaluating primary EMS against conventional or guided RCT would also be valuable for informing clinical decision‐making.

## 4. Conclusion

Within the limitations of the case reports, EMS combined with iRoot BP Plus demonstrated favorable clinical and radiographic outcomes in the management of PCO with AP following dental trauma. This approach may be considered in carefully selected cases where conventional RCT is unlikely to be predictable or associated with increased technical difficulty, as it allows direct management of periapical pathology while minimizing the risk of iatrogenic complications. Further well‐designed prospective studies are required to confirm these findings and to define the indications for primary EMS in such cases.

## Author Contributions

B.M.: patient/case selection, writing—original draft, and writing—review and editing. H.T.: writing—review and editing. J.Y.: conceptualization, methodology, investigation, supervision, and writing—review and editing.

## Funding

This study was funded by the Clinical Research Project of West China Hospital of Stomatology, Sichuan University (LCYJ‐MS‐202513).

## Disclosure

All authors approved the final version of the manuscript and agreed to be accountable for all aspects of the work.

## Ethics Statement

Ethical approval was not required for these case reports according to institutional policy.

## Consent

Written informed consent was obtained from all patients for the publication of their clinical information and accompanying images. Copies of the signed consent forms are available for review by the editor upon reasonable request.

## Conflicts of Interest

The authors declare no conflicts of interest.

## Data Availability

The data that support the findings of this study are available from the corresponding author upon reasonable request.

## References

[1] Chaniotis A. , Sousa Dias H. , and Chanioti A. , Negotiation of Calcified Canals, Journal of Clinical Medicine. (2024) 13, no. 9, 10.3390/jcm13092703, 38731233. PMC11084956 38731233

[2] Kulvitit S. , Termteerapornpimol K. , Kanpittaya P. , Prommanee S. , Thongchotchat V. , Nantanee R. , and Porntaveetus T. , Prevalence, Aetiology and Treatment Patterns of Traumatic Dental Injuries in Permanent Teeth: A Cross-Sectional Study at a Thai University Dental Hospital, Dental Traumatology. (2025) 41, no. S2, S20–S33, 10.1111/edt.70020. 41025336

[3] McCabe P. S. and Dummer P. M. H. , Pulp Canal Obliteration: An Endodontic Diagnosis and Treatment Challenge, International Endodontic Journal. (2012) 45, no. 2, 177–197, 10.1111/j.1365-2591.2011.01963.x. 21999441

[4] Jadhav G. R. and Mittal P. , Cone-Beam Computed Tomographic Scan–Based Assessment of the Correlation Between the Location of Caries and Pulp Canal Obliteration: An Aid to Treatment Planning, Journal of Endodontics. (2025) 51, no. 1, 15–20, 10.1016/j.joen.2024.10.005, 39426617. 39426617

[5] Abreu M. G. , Fernandes T. D. , Antunes L. S. , Antunes L. A. , and Faria L. C. , Prevalence of Pulp Canal Obliteration After Traumatic Dental Injuries: A Systematic Review and Meta-Analysis, Brazilian Oral Research. (2024) 38, e092, 10.1590/1807-3107bor-2024.vol38.0092. 39356901 PMC11441825

[6] Coste S. C. , Rodrigues M. A. F. , Chaves J. F. M. , Lima T. C. D. S. , Colosimo E. A. , and Bastos J. V. , A Retrospective Cohort Study of Pulp Prognosis in Luxated Permanent Teeth: A Competing Risk Analysis, Clinical Oral Investigations. (2024) 28, no. 3, 10.1007/s00784-024-05574-w, 38430349. 38430349

[7] Kevci M. , Lauridsen E. , and Andersson L. , Risk of Healing Complications Following Alveolar Process Fractures in the Primary Dentition: A Retrospective Clinical Cohort Study, Dental Traumatology. (2025) 41, no. 1, 29–36, 10.1111/edt.12992, 39318225. 39318225 PMC11733407

[8] Xavier C. B. , Rocha A. M. , Rehbien K. D. , Moura L. B. , Post L. K. , and Collares K. , Survival and Success of Teeth Involved in Alveolar Bone Injuries: Up to 10 Years of Follow-Up, Dental Traumatology. (2026) 42, no. 2, 176–186, 10.1111/edt.70012, 40874376. 40874376 PMC12990844

[9] Vinagre A. , Castanheira C. , Messias A. , Palma P. J. , and Ramos J. C. , Management of Pulp Canal Obliteration—Systematic Review of Case Reports, Medicina. (2021) 57, no. 11, 10.3390/medicina57111237, 34833455. PMC8625069 34833455

[10] Torres A. , Dierickx M. , Lerut K. , Bleyen S. , Shaheen E. , Coucke W. , Pedano M. S. , Lambrechts P. , and Jacobs R. , Clinical Outcome of Guided Endodontics Versus Freehand Drilling: A Controlled Clinical Trial, Single Arm With External Control Group, International Endodontic Journal. (2025) 58, no. 2, 209–224, 10.1111/iej.14157, 39503826. 39503826 PMC11715140

[11] Torres A. , Lerut K. , Lambrechts P. , and Jacobs R. , Guided Endodontics: Use of a Sleeveless Guide System on an Upper Premolar With Pulp Canal Obliteration and Apical Periodontitis, Journal of Endodontics. (2021) 47, no. 1, 133–139, 10.1016/j.joen.2020.09.016, 33045264. 33045264

[12] Peña-Bengoa F. , Valenzuela M. , Flores M. J. , Dufey N. , Pinto K. P. , and Silva E. J. N. L. , Effectiveness of Guided Endodontics in Locating Calcified Root Canals: A Systematic Review, Clinical Oral Investigations. (2023) 27, no. 5, 2359–2374, 10.1007/s00784-023-04863-0, 36640178. 36640178

[13] Kapoor A. , Alagarsamy R. , Lal B. , Rana A. S. , Kaur A. , Sharma S. , and Logani A. , Dynamic Navigation in Endodontics: Scope, Benefits, and Challenges - A Systematic Review, Journal of Endodontics. (2025) 51, no. 7, 879–889.e2, 10.1016/j.joen.2025.04.010, 40287086. 40287086

[14] Yang X. , Zhang Y. , Chen X. , Huang L. , and Qiu X. , Limitations and Management of Dynamic Navigation System for Locating Calcified Canals Failure, Journal of Endodontics. (2024) 50, no. 1, 96–105, 10.1016/j.joen.2023.10.010, 37890613. 37890613

[15] Wang H. , Xu X. , Bian Z. , Liang J. , Chen Z. , Hou B. , Qiu L. , Chen W. , Wei X. , Hu K. , Wang Q. , Wang Z. , Li J. , Huang D. , Wang X. , Huang Z. , Meng L. , Zhang C. , Xie F. , Yang D. , Yu J. , Zhao J. , Pan Y. , Pan S. , Yang D. , Niu W. , Zhang Q. , Deng S. , Ma J. , Meng X. , Yang J. , Wu J. , du Y. , Ling J. , Yue L. , Zhou X. , and Yu Q. , Expert Consensus on Apical Microsurgery, International Journal of Oral Science. (2025) 17, no. 1, 10.1038/s41368-024-00334-8, 39743567. PMC11693765 39743567

[16] Ng Y. L. and Gulabivala K. , Factors That Influence the Outcomes of Surgical Endodontic Treatment, International Endodontic Journal. (2023) 56, no. S2, 116–139, 10.1111/iej.13896. 36710526

[17] Zhang M. M. , Fang G. F. , Wang Z. H. , and Liang Y. H. , Clinical Outcome and Predictors of Endodontic Microsurgery Using Cone-Beam Computed Tomography: A Retrospective Cohort Study, Journal of Endodontics. (2023) 49, no. 11, 1464–1471, 10.1016/j.joen.2023.08.011, 37633517. 37633517

[18] Neelakantan P. , Vishwanath V. , Taschieri S. , and Corbella S. , Present Status and Future Directions: Minimally Invasive Root Canal Preparation and Periradicular Surgery, International Endodontic Journal. (2022) 55, no. S4, 845–871, 10.1111/iej.13750, 35426157. 35426157

[19] Eskandar R. F. , Al-Habib M. A. , Barayan M. A. , and Edrees H. Y. , Outcomes of Endodontic Microsurgery Using Different Calcium Silicate–Based Retrograde Filling Materials: A Cohort Retrospective Cone-Beam Computed Tomographic Analysis, BMC Oral Health. (2023) 23, no. 1, 10.1186/s12903-023-02782-w, 36737738. PMC9896713 36737738

[20] Dong X. , Su Q. , Li W. , Yang J. , Song D. , Yang J. , and Xu X. , The Outcome of Combined Use of iRoot BP Plus and iRoot SP for Root-End Filling in Endodontic Microsurgery: A Randomized Controlled Trial, Clinical Oral Investigations. (2024) 28, no. 3, 10.1007/s00784-024-05569-7, 38430316. 38430316

[21] Falcon P. A. , Falcon C. Y. , Abbasi F. , and Hirschberg C. S. , Chamberless Endodontic Access for Treatment of Calcified Anterior Central Incisors, Journal of Endodontics. (2021) 47, no. 2, 322–326, 10.1016/j.joen.2020.10.017, 33129898. 33129898

[22] Nagendrababu V. , Chong B. S. , McCabe P. , Shah P. K. , Priya E. , Jayaraman J. , Pulikkotil S. J. , Setzer F. C. , Sunde P. T. , and Dummer P. M. H. , PRICE 2020 Guidelines for Reporting Case Reports in Endodontics: A Consensus-Based Development, International Endodontic Journal. (2020) 53, no. 5, 619–626, 10.1111/iej.13285, 32090342. 32090342

[23] Kim S. and Kratchman S. , Modern Endodontic Surgery Concepts and Practice: A Review, Journal of Endodontics. (2006) 32, no. 7, 601–623, 10.1016/j.joen.2005.12.010, 16793466. 16793466

[24] Wei X. , Du Y. , Zhou X. , Yue L. , Yu Q. , Hou B. , Chen Z. , Liang J. , Chen W. , Qiu L. , and Huang X. , Expert Consensus on Digital Guided Therapy for Endodontic Diseases, International Journal of Oral Science. (2023) 15, no. 1, 10.1038/s41368-023-00261-0, 38052782. PMC10697975 38052782

[25] Patel S. , Brown J. , Pimentel T. , Kelly R. D. , Abella F. , and Durack C. , Cone Beam Computed Tomography in Endodontics – A Review of the Literature, International Endodontic Journal. (2019) 52, no. 8, 1138–1152, 10.1111/iej.13115. 30868610

[26] Sabeti M. , Gabbay J. , and Ai A. , Endodontic Surgery and Platelet Concentrates: A Comprehensive Review, Periodontology 2000. (2025) 97, no. 1, 308–319, 10.1111/prd.12593, 39135355. 39135355

[27] Yan L. , Lin J. , Yang L. , He S. , Tan X. , and Huang D. , Clinical Effect Evaluation of Concentrated Growth Factor in Endodontic Microsurgery: A Cross-Sectional Study, Journal of Endodontics. (2023) 49, no. 7, 836–845, 10.1016/j.joen.2023.05.005, 37182792. 37182792

[28] Arias A. and Peters O. A. , Present Status and Future Directions: Canal Shaping, International Endodontic Journal. (2022) 55, no. S3, 637–655, 10.1111/iej.13698, 35118683. 35118683 PMC9303733

[29] Boutsioukis C. and Arias-Moliz M. T. , Present Status and Future Directions – Irrigants and Irrigation Methods, International Endodontic Journal. (2022) 55, no. S3, 588–612, 10.1111/iej.13739, 35338652. 35338652 PMC9321999

[30] Zou X. , Zheng X. , Liang Y. , Zhang C. , Fan B. , Liang J. , Ling J. , Bian Z. , Yu Q. , Hou B. , Chen Z. , Wei X. , Qiu L. , Chen W. , He W. , Xu X. , Meng L. , Zhang C. , Chen L. , Deng S. , Lei Y. , Xie X. , Wang X. , Yu J. , Zhao J. , Shen S. , Zhou X. , and Yue L. , Expert Consensus on Irrigation and Intracanal Medication in Root Canal Therapy, International Journal of Oral Science. (2024) 16, no. 1, 10.1038/s41368-024-00280-5, 38429299. PMC10907616 38429299

[31] Camilleri J. , Atmeh A. , Li X. , and Meschi N. , Present Status and Future Directions: Hydraulic Materials for Endodontic Use, International Endodontic Journal. (2022) 55, no. S3, 710–777, 10.1111/iej.13709, 35167119. 35167119 PMC9314068

[32] Wang C. , Chen S. , Wang L. , Li B. , Bai M. , Yu F. , and Ye L. , A Randomized Controlled Trial Comparing Novel and Traditional Apical Barrier Techniques in Endodontic Procedures, Clinical Oral Investigations. (2025) 29, no. 7, 10.1007/s00784-025-06450-x, 40616689. 40616689

